# Prevalence of cervical cancer and its associated factors among women living with HIV attending public referral hospitals in Amhara Region, Ethiopia, 2025, a cross-sectional study

**DOI:** 10.1186/s12879-026-12976-6

**Published:** 2026-02-28

**Authors:** Muluken Belachew Mengistie, Wubete Lule Ayalew, Simane Kefale Mengistie, Ayenew Sisay Gebeyehu, Sefefe Birhanu Tizie, Ashagrie Anteneh Mekonen, Mezigebu Lule Ayalew

**Affiliations:** 1 Department of Health Informatics, College of Medicine and Health Science, Debre Markos University, Debre Markos, Ethiopia; 2 Department of Midwifery, College of Medicine and Health Science, Debre Markos University, Debre Markos, Ethiopia; 3 Department of Gynecology and Obstetrics, School of Medicine, College of Medicine and Health Science, University of Gondar, Gondar, Ethiopia

**Keywords:** Cervical cancer, Prevalence, Factors, Women, and Human immunodeficiency virus

## Abstract

**Introduction:**

Cervical cancer remains a leading cause of morbidity and mortality among women living with HIV worldwide, particularly in developing countries. In Ethiopia, this population faces a substantial burden of cervical cancer, marked by high rates of precancerous cervical lesions and low uptake of screening services. Therefore, this study aims to determine the prevalence of cervical cancer and identify its associated factors among women living with HIV attending referral hospitals in the Amhara Region, Northwest Ethiopia, to inform effective prevention and control strategies.

**Methods:**

An institution-based cross-sectional study was conducted among women living with HIV who attended selected referral hospitals in the Amhara Region, Northwest Ethiopia, from August to October 2025. Study participants were selected using a systematic random sampling technique. Data were collected using a pretested, interviewer-administered questionnaire. The collected data were entered and analyzed using SPSS software. Descriptive statistics were used to summarize the data, while binary logistic regression analysis was employed to assess the prevalence of cervical cancer and identify associated factors. Statistical significance was determined at a p-value of less than 0.05.

**Results:**

Among the total study participants, 48 (15.5%) of women living with HIV were diagnosed with cervical cancer. In the final analysis, the number of sexual partners and a history of sexually transmitted infections were found to be significantly associated with cervical cancer. Women with multiple sexual partners had higher odds of developing cervical cancer [AOR = 2.41; 95% CI: 1.49–4.07], and those with a history of sexually transmitted infections were also at increased risk [AOR = 2.92; 95% CI: 1.28–3.12].

**Conclusion:**

This study found that the overall prevalence of cervical cancer among women living with HIV attending referral hospitals in the Amhara region was high. Having multiple sexual partners and a history of sexually transmitted infections were associated with an increased likelihood of developing cervical cancer. Therefore, promoting monogamous sexual partnerships and preventing sexually transmitted infections are key strategies to protect women living with HIV from cervical cancer.

**Supplementary Information:**

The online version contains supplementary material available at 10.1186/s12879-026-12976-6.

## Introduction

Cervical cancer is the second most common malignancy among women worldwide, following breast cancer, and remains the leading cause of gynecological cancer–related mortality in developing countries [1]. Globally, more than 530,000 new cases and over 270,000 deaths are reported each year, with approximately 85% of cases occurring in low-income countries due to limited awareness and inadequate screening practices [2]. The disease arises from the uncontrolled proliferation of abnormal cells in the cervix, which may invade surrounding tissues and metastasize to distant organs [3]. Early detection through screening enables identification of precancerous lesions, making cervical cancer one of the most preventable and treatable forms of cancer when diagnosed at an early stage [4]. The human papillomavirus (HPV), which is also the most prevalent sexually transmitted infection, is a necessary cause of cervical cancer [4].

Women living with human immunodeficiency virus (WLHIV) are disproportionately affected by cervical cancer due to compromised immune function, which increases their susceptibility to HPV infection and the persistence of high-risk HPV types that drive cervical carcinogenesis [5]. Immunosuppression caused by Human Immunodeficiency Virus (HIV) accelerates progression from HPV infection to precancerous lesions and invasive cervical cancer, resulting in incidence rates that are several times higher than in HIV-negative women [6]. Studies have consistently shown that WLHIV have a heightened risk of developing cervical precancerous lesions and cancer, emphasizing the importance of targeted screening and early detection in this vulnerable population [7].

In Ethiopia, cervical cancer is a leading cause of cancer-related morbidity and mortality among women. Although exact national incidence estimates vary, thousands of new cases are detected annually, and many women present at advanced stages with poor prognoses [8]. Despite the adoption of the World Health Organization’s cervical cancer prevention strategy by the Ethiopian Ministry of Health, which recommends regular screening and timely treatment, screening utilization remains low among women living with HIV who are at increased risk [9, 10].

Key epidemiological risk factors for cervical cancer include multiple sexual partners, early initiation of sexual activity, persistent infection with HPV, lower genital tract neoplasia, sexual contact with partners who have cervical neoplasia, a history of sexually transmitted infections, cigarette smoking, HIV infection, other forms of immunosuppression, and prolonged use of oral contraceptive pills [11]. Substantial reductions in cervical cancer incidence and mortality have been achieved in developed countries through organized and systematic cytological screening programs. These findings demonstrate that cervical cancer is largely preventable when effective screening strategies are implemented. However, in many resource-limited settings, such reductions have not been realized, primarily due to the absence or limited availability of systematic screening services [12].

HIV infection continues to pose a major global public health challenge, affecting millions of people worldwide. Sub-Saharan Africa carries the greatest burden of the epidemic, and Ethiopia is among the countries significantly affected. Spatial analyses conducted within Ethiopia have consistently identified high-prevalence clusters of HIV in Addis Ababa and surrounding areas, including parts of the Afar, Tigray, and Amhara regional states, as well as central Oromia [13]. HIV infection is a well-established risk factor for cervical cancer, largely due to its interaction with HPV, which influences both the development and progression of the disease [14]. WLWH exhibit some of the highest reported rates of HPV infection, and HIV adversely affects the natural history of HPV by increasing persistence and progression to precancerous and cancerous lesions [14]. Among WLWH, cervical cancer is the most commonly diagnosed malignancy and is recognized as an AIDS-defining condition [15].

Evidence from studies in Ethiopia indicates that cervical cancer significantly affects women’s quality of life, highlighting the importance of regular screening [16]. Implementing primary prevention measures, including HPV vaccination, alongside expanded screening programs, is critical to reducing the morbidity, mortality, and economic burden associated with the disease [17]. Despite these efforts, Ethiopian women continue to die from preventable conditions such as cervical cancer. Achieving a meaningful reduction in incidence and mortality requires not only widespread HPV vaccination but also the strengthening of effective screening and prevention services to ensure early detection and timely treatment. Understanding the prevalence of cervical cancer and its associated factors is therefore essential, particularly among women living with HIV, who are at higher risk. In line with this, the present study aims to assess the prevalence of cervical cancer and identify associated factors among women living with HIV attending referral hospitals in the Amhara Region, Northwest Ethiopia.

## Methods

### Study design and setting

An institution-based cross-sectional study was conducted from August to October 2025 among women living with HIV attending selected referral hospitals in Amhara Region, Northwest Ethiopia. The study utilized cervical cancer data from five referral hospitals in a region: Debre Markos, Finote Selam, Felege Hiwot, Gondar, and Dessie hospitals. The hospitals provide comprehensive health services, including inpatient and outpatient care, maternal and child health services, specialized care, and tertiary-level referral treatment.

### Source and study population

The source population comprised all women living with HIV attending selected referral hospitals in the Amhara Region, Northwest Ethiopia. The study population included all sampled women living with HIV who attended these hospitals during the study period. For patients with multiple visits, only data from their first visit were included in the analysis to ensure that each patient was counted once.

### Sample size determination

The sample size for the quantitative study is determined using a single population proportion formula.

$$\rm n= (Z\alpha/2)^2\:p (1-p)/ d^2$$

Where: Z = Standard Normal deviation (1.96 for a 95% confidence level).

n = the sample size

p = proportion of the population (take p value 0.24 from the previous study [18]

d=margin of error (d = 0.05)

Sample size (n) = 280, with a non-response rate (10%) = 308 respondents

### Sampling technique and procedure

All eligible hospitals in the Amhara Region were identified from the official list obtained from the Regional Health Bureau. Eligibility was determined based on predefined criteria, specifically the level of care, with only referral hospitals included in the sampling frame. From these, referral hospitals were selected using a simple random sampling technique to ensure representativeness and minimize selection bias.

**Fig. 1:**
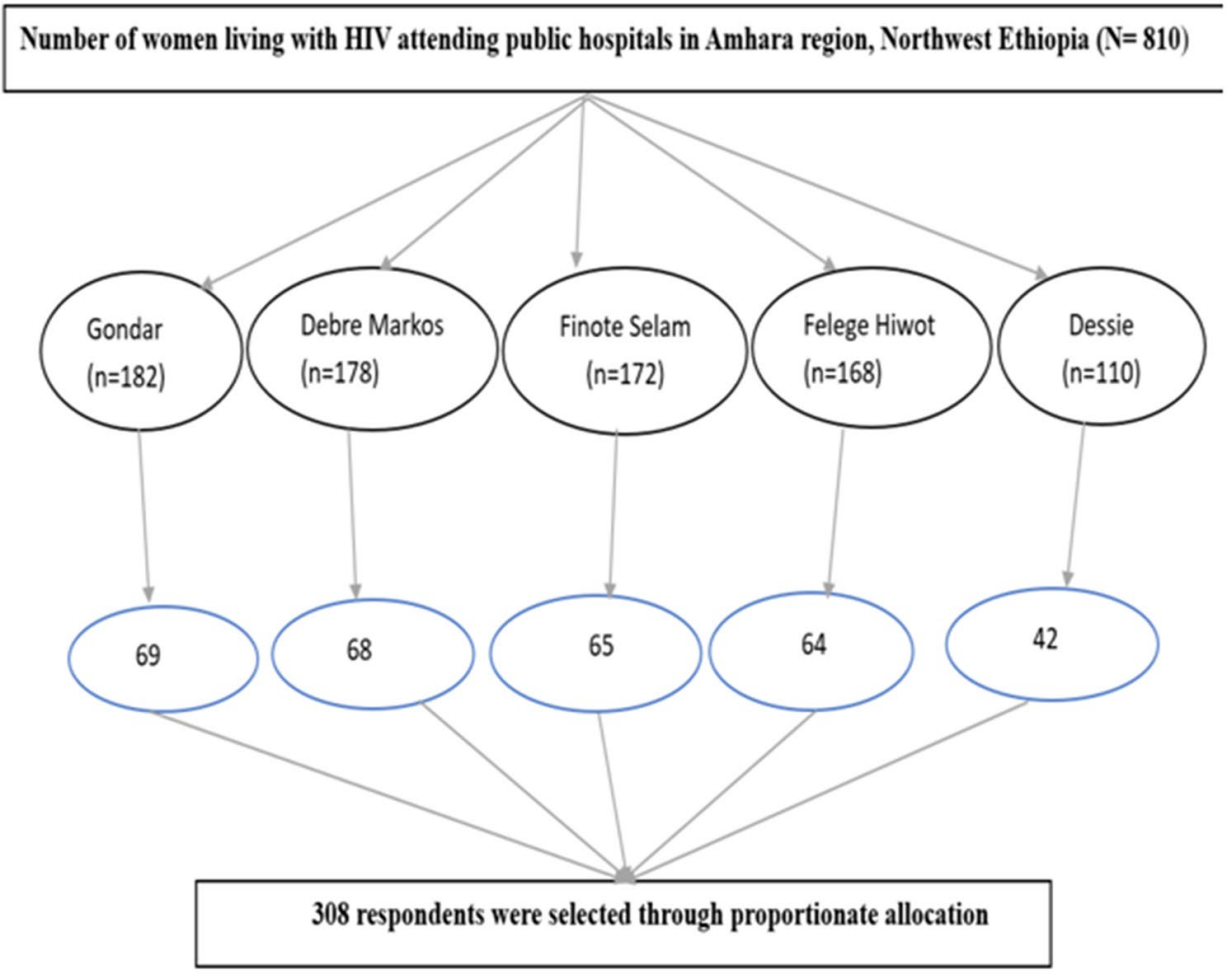
Sampling procedure for this study

The sampling frame for selecting study participants consisted of a complete list of all women living with HIV who were receiving care at the selected referral hospitals during the study period. This list was obtained from the patient registry or medical records at each hospital. A systematic random sampling technique was used to select study participants from the total women living with HIV attending selected referral hospitals in Amhara Region, Northwest Ethiopia. Currently, there are a total of 810 women living with HIV attending the selected referral hospitals in Amhara Region, and from the total, 308 women were selected through proportionate allocation, and the selected patients were addressed through an interviewer-administered questionnaire (Fig. 1).

### Eligibility criteria

All women living with HIV who attended selected referral hospitals in the Amhara Region, Northwest Ethiopia, were considered for inclusion in the study. In contrast, Women living with HIV who were unable to provide a response due to serious illness at the time of data collection were excluded from the study.

### Study variables

The dependent variable of this study was cervical cancer. The independent variables included socio-demographic factors (age, educational status, marital status, occupation, monthly household income, and place of residence); reproductive health–related factors (number of liveborn children, current number of sexual partners, history of sexually transmitted infections, age at first sexual intercourse, and family planning history); and behavioral factors (smoking and alcohol consumption).

### Data collection techniques and procedures

A pretested, interviewer-administered Amharic version of the questionnaire was used to collect data on the prevalence of cervical cancer and its associated factors. The questionnaire, which included sections on socio-demographic, reproductive health–related, and behavioral factors, was adapted from an extensive review of relevant literature and previously published studies [18, 19]. Data collection was carried out by five bachelor’s degree–level nurses, supervised by three trained supervisors. During the data collection process, data collectors introduced themselves and their research assistants to the selected respondents. The consent form was read aloud to each participant, after which they decided whether to take part in the study. Only those who provided informed consent were administered the pretested, structured questionnaire.

### Data quality assurance

Before conducting the main study, a pretest was performed on 10% of the sample at Injibara Hospital to assess the effectiveness and representativeness of the survey. Based on the pretest findings, data collectors were reoriented, and the questionnaire was revised as needed. The completeness and consistency of the data were regularly monitored throughout the study. To ensure high data quality, both data collectors and supervisors received two days of training before data collection, and timely supervision was maintained during the data collection process.

### Measurement

Cervical cancer screening for women living with HIV at selected referral hospitals in the Amhara Region was conducted by trained healthcare professionals (nurses and midwives) as part of routine services integrated into HIV care clinics during the study period (August to October 2025). Screening was primarily performed based on clinical assessment using visual inspection with acetic acid (VIA) at the HIV care clinics, where eligible HIV-positive women received care.

### Data processing and analysis

The collected data were manually coded and cleaned before being entered into the software for analysis. The questionnaire data were then entered and analyzed using SPSS software. Descriptive statistics were employed to summarize the socio-demographic characteristics of women living with HIV. A binary logistic regression model was used to identify factors associated with cervical cancer, followed by multivariable analysis to determine independent associations, with a significance level set at *P* < 0.05. Results were interpreted using AOR with 95% CI.

### Operational definitions

#### Cervical cancer

Cervical cancer is a type of cancer in which the cells of the cervix develop abnormally and form a tumor [20].

#### Smoking

a smoker is a person who has smoked during the past 12 months and smokes currently either every day or sometimes [21].

## Results

### Socio-demographic characteristics of women living with HIV

A total of 308 women living with HIV participated in this study. From the total, more than half of the participants (59.7%) were in the age group of ≥ 50 years. Majorities (52%) of the respondents were married, and 38.6% had higher education, followed by secondary education (20.1%). In terms of occupation, 24.1% of respondents were Government employees, followed by housewives (23.7%) and non-government employees (21.4%). In addition, the majority of women (63.3%) were rural residents, and 57.1% of participants had a monthly income of ≥ 3000 ETB (Table 1).

**Table 1:** Socio-demographic information of women living with HIV attending public referral hospitals in Amhara Region, 2025

Variable	Category	Frequency (#)	Percent (%)
Age (years)	18–25	36	11.7%
	26–35	37	12.0%
	36–49	51	16.6%
	≥ 50	184	59.7%
Marital status	Single	46	14.9%
	Married	160	52%
	Divorced	27	8.8%
	Separated	14	4.5%
	Widowed	61	19.8%
Educational status	Unable to read and write	59	19.2%
	Able to read and write	45	14.6%
	Primary education	23	7.5%
	Secondary education	62	20.1%
	Higher education	119	38.6%
Occupation	Government employee	74	24.1%
	Non-government employee	66	21.4%
	Farmer	23	7.5%
	Merchant	36	11.7%
	Daily laborer	14	4.5%
	Student	9	2.9%
	Unemployed	13	4.2%
	House wife	73	23.7%
Place of residence	Urban	113	36.7%
	Rural	195	63.3%
Monthly household income	< 1000	27	8.8%
	1000–1999	45	14.6%
	2000–2999	60	19.5%
	≥ 3000	176	57.1%

### Cervical cancer prevalence

**Fig. 2:**
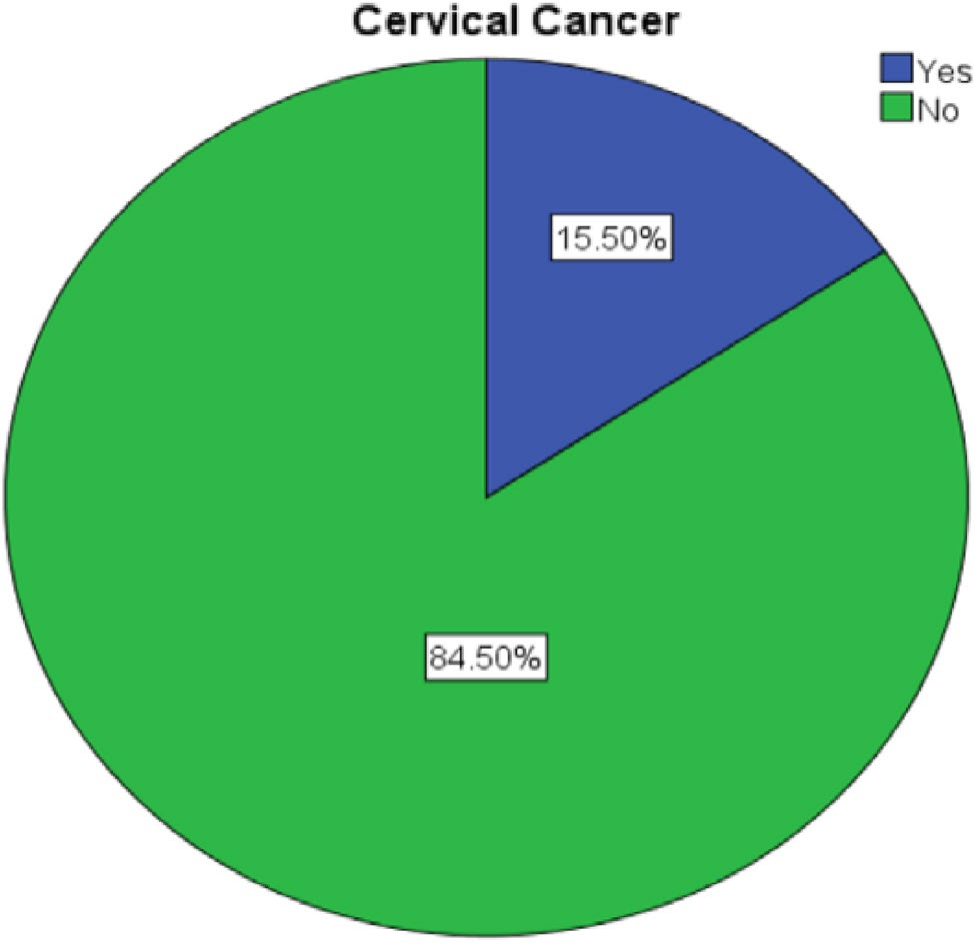
Prevalence of cervical cancer among women living with HIV attending public referral hospitals in Amhara Region, Northwest Ethiopia, 2025

Among the total respondents, 48 (15.5%) of women living with HIV were found to have cervical cancer (Fig. 2).

### Factors associated with cervical cancer

In the final multivariable logistic regression model, the number of sexual partners and a history of sexually transmitted infections were identified as significant factors associated with cervical cancer among women living with HIV. This study found that women living with HIV who had multiple sexual partners had 2.41 times higher odds of developing cervical cancer compared with those who had a single sexual partner (AOR = 2.41; 95% CI: 1.49–4.07). In addition, women with a history of sexually transmitted infections were 3 times more likely to develop cervical cancer than those patients without a prior history of STIs (AOR = 2.92; 95% CI: 1.28–3.12) (Table 2).

**Table 2:** Bivariable and multivariable analysis of factors associated with cervical cancer among women living with HIV attending public referral hospitals in Amhara Region, Northwest Ethiopia, 2025

Variables	Cervical Cancer (*n* = 308)		COR (95% CI)	AOR (95% CI)
	Yes	No		
**Age (years)**				
18–25	7 (19.4%)	29 (80.6%)	1	1
26–35	3 (8.1%)	34 (91.9%)	2.5 (1.02, 6.03)	0.8 (0.27, 2.61)
36–49	10 (19.6%)	41 (80.4%)	6.8 (1.99, 23.12)	2.9 (0.77, 11.43)
≥50	65 (35.3%)	119 (64.7%)	2.5 (1.14, 5.28)	1.4 (0.52, 3.62)
**Educational status**				
Unable to read and write	27 (45.8%)	32 (54.2%)	1	1
Able to read and write	11 (24.4%)	34 (75.6%)	0.3 (0.12, 0.57)	0.33 (0.14, 1.95)
Primary education	4 (17.4%)	19 (82.6%)	0.7 (0.29, 1.67)	0.74 (0.25, 2.26)
Secondary education	18 (29%)	44 (71%)	1.1 (0.32, 1.58)	1.03 (0.22, 5.01)
Higher education	25 (21%)	94 (79%)	0.5 (0.25, 1.19)	0.55 (0.21, 1.46)
**Place of residence**				
Urban	9 (8%)	104 (92%)	1	1
Rural	76 (39%)	119 (61%)	8.3 (3.91, 17.56)	5.29 (0.35, 11.87)
**Monthly household income**				
<1000	5 (18.5%)	22 (81.5%)	1	1
1000–1999	8 (17.8%)	37 (82.2%)	2.18 (0.77, 6.12)	1.775 (0.48, 6.58)
2000–2999	16 (26.7%)	44 (73.3%)	2.29 (0.99, 5.31)	2.046 (0.72, 5.85)
≥3000	56 (31.8%)	120 (68.2%)	1.36(0.69, 2.67)	1.431 (0.61, 3.38)
**No of sexual partners (Current)**				
Single	16 (39.0%)	25 (61.0%)	1	1
Multiple	59 (23%)	198 (77%)	2.54 (1.27, 3.08)	2.41 (1.49, 4.07) ^*^
**History of STI**				
No	46 (18.8%)	199 (81.2%)	1	1
Yes	39 (60.0%)	26 (40.0%)	2.14 (1.12, 3.12)	2.92 (1.28, 3.12) ^*^
**Age at first intercourse (Years)**				
<18	21 (37.5%)	35 (62.5%)	1	1
>=18	64 (25.4%)	188 (74.6%)	0.55 (0.30, 1.04)	1.07 (0.42, 2.71)
**Smoking**				
No	61 (25.6%)	177 (74.4%)	1	1
Yes	24 (34.3%)	46 (65.7%)	1.21 (0.72, 2.32)	1.51(0.51, 2.52)

^1=Reference category * Significant association^

## Discussion

This study found that the overall prevalence of cervical cancer among women living with HIV was 48 (15.5%). This result is comparable to findings from studies conducted in Ethiopia (15.34%) [22], Southwest Ethiopia (18.7%) [23], Malawi (12.4%), and Nigeria (16%) [24]. However, the prevalence reported in this study is higher than that observed in Madagascar (11.3%) [24] and the Mekelle zone, Northern Ethiopia (6.7%) [10]. Conversely, it is lower than the prevalence reported in studies from Bugando Medical Centre, Mwanza, Tanzania (26.8%) [25], and Gandhi, Ethiopia (23.5%) [18]. The observed variations in cervical cancer prevalence across studies may be explained by differences in HIV-related factors, including the duration of HIV infection, degree of immunosuppression as indicated by CD4 cell count, antiretroviral therapy coverage, and treatment adherence, all of which affect the persistence of HPV infection and its progression to cervical cancer [26]. Additionally, disparities in access to and utilization of cervical cancer screening services, availability of trained healthcare professionals, and the screening methods employed—such as visual inspection with acetic acid, Pap smear may further contribute to the observed differences in prevalence [27].

This study found that women living with HIV who had multiple sexual partners were more than twice as likely to develop cervical cancer compared to women with a single sexual partner (AOR = 2.41, 95% CI: 1.49–4.07). This finding is consistent with evidence from a study conducted at Bugando Medical Center, Tanzania, which reported a similar association between multiple sexual partnerships and cervical cancer risk [25]. Likewise, a recent study in Ethiopia demonstrated that women with multiple sexual partners had an increased likelihood of developing cervical cancer [21]. The elevated risk is primarily attributed to increased exposure to persistent HPV infections, the main causative agent of cervical cancer, with HIV-positive women being particularly vulnerable due to immune suppression [28]. Supporting this association, a cross-sectional study at Yirgalem Hospital in 2017 found that women with multiple sexual partners were 40 times more likely to develop cervical cancer compared to women with a single partner (AOR = 40; 95% CI: 22.8–70) [28]. Similarly, a retrospective cross-sectional study conducted at Gandhi Hospital in 2021 reported that women with multiple sexual partners had nearly twice the risk of developing cervical cancer (AOR = 1.83; 95% CI: 1.21–3.29) [18].

This study also found that women with a history of STI were 3 times more likely to develop cervical cancer compared to those without a history of STI (AOR = 2.92; 95% CI: 1.28, 3.12). This finding aligns with a study conducted in Ethiopia in 2024, which reported that women with a history of STI had more than three times higher odds of developing precancerous cervical lesions compared to women without such a history (AOR = 3.12; 95% CI: 1.38–7.05) [22]. Similarly, evidence from Bugando Medical Center in Tanzania demonstrated that a prior STI acts as a key cofactor in the progression of human HPV infection to cervical dysplasia and invasive cervical cancer [25]. Studies conducted in Yirgalem and Gandhi hospitals also support the significant association between a history of STI and cervical cancer risk [18, 28]. Chronic inflammation resulting from STIs creates a biological environment that promotes persistent HPV infection and facilitates the malignant transformation of cervical cells [29]. In women living with HIV, this risk is further amplified, as immunosuppression impairs the body’s ability to control HPV replication and expression, accelerating the development of cervical intraepithelial lesions and increasing the likelihood of progression to cervical cancer [29]. STIs can increase the risk of cervical cancer among women living with HIV by promoting persistent HPV infection and facilitating progression to precancerous and cancerous lesions. HPV infection is the principal cause of cervical cancer, and its persistence is central to cervical carcinogenesis [30]. Co-existing STIs such as *Chlamydia trachomatis* or other genital infections may produce chronic local inflammation, disrupt the cervical epithelium, and alter immune responses, creating an environment that promotes HPV acquisition and persistence [31]. Among women living with HIV, immunosuppression further impairs the ability to clear HPV infection, resulting in prolonged infection and accelerated progression from precancerous lesions to invasive cervical cancer [32].

## Conclusion

This study found that the overall prevalence of cervical cancer among women living with HIV attending public referral hospitals in the Amhara region was high. Having multiple sexual partners and a history of sexually transmitted infections were associated with an increased likelihood of developing cervical cancer among women living with HIV attending public referral hospitals in Amhara Region. However, the study was conducted in public referral hospitals, and data were collected from only five referral hospitals, which may limit the generalizability of the findings to all women living with HIV in the region and may not fully capture regional variations in cervical cancer prevalence or associated factors. Therefore, future studies should include a larger number of hospitals, including both public and private facilities across different areas of the Amhara Region, to improve generalizability and better capture regional variations in cervical cancer prevalence and its associated factors among women living with HIV.

## Supplementary Information

Below is the link to the electronic supplementary material.

Supplementary Material 1

## Data Availability

All relevant data are included within the manuscript and can also be obtained from the corresponding author.

## Abbreviations

AOR: Adjusted Odds Ratio
CI: Confidence Interval
CD4: Cluster of Differentiation 4
COR: Crude Odds Ratio
HIV: Human Immunodeficiency Virus
HPV: Human Papilloma Virus
SPSS: Statistical Package for Social Science
STI: Sexually Transmitted Infection
WLWH: Women living with HIV

## References

[1] Teka T, et al. Magnitude and factors associated with precervical cancer among screened women in Southern Ethiopia. 2019;2019(1): p. 5049752.

[2] Hailemariam G, et al. Magnitude and associated factors of VIA positive test results for cervical cancer screening among refugee women aged 25–49 years in North Ethiopia. 2020. 20(1): p. 858. 10.1186/s12885-020-07344-9 PMC7487853 32894100

[3] Weldegebreal F, Worku TJCC. Precancerous cervical lesion among HIV-positive women in Sub-Saharan Africa: a systematic review and meta-analysis. 2019;26(1):p. 1073274819845872. 10.1177/1073274819845872 PMC6572896 31043067

[4] Emru K, Abebaw T-A, Abera AJWsH. Role of awareness on cervical cancer screening uptake among HIV positive women in Addis Ababa, Ethiopia: a cross-sectional study. 2021;17: p. 17455065211017041. 10.1177/17455065211017041 PMC8188969 34096400

[5] Denny L, et al. Interventions to close the divide for women with breast and cervical cancer between low-income and middle-income countries and high-income countries. 2017;389(10071):pp. 861–70. 10.1016/S0140-6736(16)31795-0 27814963

[6] Liu G, et al. HIV-positive women have higher risk of human papilloma virus infection, precancerous lesions, and cervical cancer. 2018;32(6):pp. 795–808. 10.1097/QAD.0000000000001765 PMC5854529 29369827

[7] Kelly H, et al. Association of antiretroviral therapy with high-risk human papillomavirus, cervical intraepithelial neoplasia, and invasive cervical cancer in women living with HIV: a systematic review and meta-analysis. 2018;5(1):pp. e45–58. 10.1016/S2352-3018(17)30149-2 PMC5757426 29107561

[8] Mosquera I, et al. Cancer burden and status of cancer control measures in fragile states: a comparative analysis of 31 countries. 2022;10(10):pp. e1443–52. 10.1016/S2214-109X(22)00331-X PMC9638035 36113529

[9] Tesfaye D, et al. Cervical cancer screening uptake and associated factors among women living with human immunodeficiency virus in public hospitals, Eastern Ethiopia. 2023;13:p. 1249151. 10.3389/fonc.2023.1249151 PMC10642187 37965474

[10] Bayu H, et al. Cervical cancer screening service uptake and associated factors among age eligible women in Mekelle Zone, Northern Ethiopia, 2015: a community based study using health belief model. 2016;11(3):p. e0149908. 10.1371/journal.pone.0149908 PMC4786115 26963098

[11] Goodwin T, et al. Current diagnosis and treatment obstetrics and gynecology. McGraw-Hill Medical; 2012.

[12] Franco E. L.J.J.o.t.N.C.I., Persistent HPV infection and cervical cancer risk: is the scientific rationale for changing the screening paradigm enough? Oxford University Press; 2010;pp. 1451–3. 10.1093/jnci/djq357 20841606

[13] Getiye DK, G.D.K., et al. Trends and spatial distributions of HIV prevalence in Ethiopia. 2019. 10.1186/s40249-019-0594-9 PMC6796490 31623689

[14] Stelzle D, et al. Estimates of the global burden of cervical cancer associated with HIV. 2021;9(2):pp. e161–9. 10.1016/S2214-109X(20)30459-9 PMC7815633 33212031

[15] Beas-Lozano EL, et al. Current management of cervical cancer in women living with HIV. 2024;204:p. 104519. 10.1016/j.critrevonc.2024.104519 39322024

[16] Bayisa K, et al. Cervical cancer screening utilization and associated factors among women living with HIV in South West Ethiopia. 2025;15(1):p. 35081. 10.1038/s41598-025-18987-8 PMC12508089 41062593

[17] Hailu A, D.H.J.B.c. Mariam, Patient side cost and its predictors for cervical cancer in Ethiopia: a cross sectional hospital based study. 2013;13(1):p. 69. 10.1186/1471-2407-13-69 PMC3576296 23391288

[18] Mekuria M, et al. Prevalence of cervical cancer and associated factors among women attended cervical cancer screening center at Gahandi Memorial Hospital. Ethiopia. 2021;20:11769351211068431. 10.1177/11769351211068431 PMC8725021 34992337

[19] Hailemariam T, et al. Prevalence of cervical cancer and associated risk factors among women attending cervical cancer screening and diagnosis center at Yirgalem General Hospital, Southern Ethiopia. 2017;9(11):pp. 730–5.

[20] Sung H, et al. Global cancer statistics 2020: GLOBOCAN estimates of incidence and mortality worldwide for 36 cancers in 185 countries. 2021;71(3):pp. 209–49. 10.3322/caac.21660 33538338

[21] Paczesny S, et al. National Institutes of Health consensus development project on criteria for clinical trials in chronic graft-versus-host disease: III. The 2014 Biomarker Working Group Report. 2015;21(5):pp. 780–92. 10.1016/j.bbmt.2015.01.003 PMC4408233 25644957

[22] Ferede YA, Tassew WC, A.M.J.B.c. Zeleke, Precancerous cervical lesion and associated factors among HIV-infected women in Ethiopia: systematic review and meta-analysis. 2024;24(1) p. 678. 10.1186/s12885-024-12462-9 PMC11149367 38831404

[23] Lemu LG, et al. Precancerous cervical lesions among HIV-Infected women attending HIV Care and Treatment clinics in Southwest Ethiopia: a cross-sectional study. 2021:pp. 297–303. 10.2147/IJWH.S295137 PMC7937384 33688268

[24] Organization WH, I.A.f.R.o. Cancer, Prevention of cervical cancer through screening using visual inspection with acetic acid (VIA) and treatment with cryotherapy. A demonstration project in six African countries: Malawi, Madagascar, Nigeria, Uganda, the United Republic of Tanzania, and Zambia. 2012.

[25] Kafuruki L, et al. Prevalence and predictors of cervical intraepithelial neoplasia among HIV infected women at Bugando Medical Centre. Mwanza-Tanzania. 2013;8(1):45. 10.1186/1750-9378-8-45 PMC3833176 24228805

[26] Lin C, Franceschi S, MJTLID. Clifford G. Human papillomavirus types from infection to cancer in the anus, according to sex and HIV status: a systematic review and meta-analysis. 2018;18(2):pp. 198–206. 10.1016/S1473-3099(17)30653-9 PMC5805865 29158102

[27] Msyamboza KP, et al. Cervical cancer screening uptake and challenges in Malawi from 2011 to 2015: retrospective cohort study. 2016;16(1):p. 806. 10.1186/s12889-016-3530-y PMC4989288 27535359

[28] Layet F, et al. Factors associated with utilization of cervical cancer screening services among HIV-positive women aged 18 to 49 years at Lira regional referral hospital. North Uganda. 2024;24(1):114. 10.1186/s12905-024-02957-9 PMC10863236 38347497

[29] Pella-Saavedra P, et al. Prevalence of coinfections in a cross-sectional cohort of women screened for multiple pathogens in Peru. 2023;9(3). 10.1016/j.heliyon.2023.e14257 PMC10025105 36950601

[30] Latorre-Millán M, et al. HPV-associated sexually transmitted infections in cervical Cancer screening: a prospective cohort study. 2025;17(2):p. 247. 10.3390/v17020247 PMC11861167 40007002

[31] Ray, A.J.I.J.o.M.S. Human papillomavirus and other relevant issues in cervical cancer pathogenesis. 2025;26(12):5549. 10.3390/ijms26125549 PMC12193582 40565012

[32] Swase TD, et al. The impact of HPV/HIV co-infection on immunosuppression, HPV genotype, and cervical cancer biomarkers. 2025;25(1):p. 202. 10.1186/s12885-025-13516-2 PMC11796042 39910495

