# Supplementary material for: Prevalence of cervical cancer and its associated factors among women living with HIV attending public referral hospitals in Amhara Region, Ethiopia, 2025, a cross-sectional study
Source: BMC Infect Dis. 2026 Feb 28;26:722. doi: 10.1186/s12879-026-12976-6 (PMC13059549; doi:10.1186/s12879-026-12976-6)
Supplement: Supplementary file 1 — Supplementary Material 1 [file 12879_2026_12976_MOESM1_ESM.docx]

**English Version Questionnaire for the Study**

**Research Title: “Prevalence of Cervical Cancer and Its Associated Factors Among Women Living with HIV Attending Public Hospitals in Amhara Region, Ethiopia, 2025, a cross-sectional study”**

| **S/No** | **Questions** | | | | **Category/Code** | | |
| --- | --- | --- | --- | --- | --- | --- | --- |
| **Part 1: Socio-demographic information assessment questions** | | | | | | | |
| 100 | Age in years | | | ________ years. | | | |
| 101 | Marital status | | | 1. Single  2. Married  3. Divorced  4. Separated  5. Widowed | | | |
| 102 | Educational status | | | 1. Unable to read and write  2. Able to read and write  3. Primary education (1-8 grade)  4. Secondary education (9-12 grade)  5. Higher education | | | |
| 103 | Occupation | | | 1. Employed  2. Unemployed | | | |
| 104 | Place of residence | | | 1. Urban  2. Rural | | | |
| 105 | Monthly Household Income | | | _______________________ETB. | | | |
| **Part 2: Cervical Cancer assessment question** | | | | | | | |
| 200 | Do you have a confirmed cervical cancer? | | | | | | 1. Yes  2. No |
| **Part 3: Reproductive health-related variables assessment questions** | | | | | | | |
| 300 | | Number of liveborn children you have delivered? | | | | ­­­­­­­­­­­­­­­­__________________. | |
| 301 | | Age at 1^st^ intercourse | | | | __________________Years. | |
| 302 | | Number of sexual partners | | | | 1. One  2. Multiple | |
| 303 | | Sexually transmitted infection history | | | | 1. Yes  2. No | |
| 304 | | Family planning history | | | | 1. Yes  2. No | |
| **Part 4: Behavioral factors assessment questions** | | | | | | | |
| 400 | | | Are you currently a cigarette smoker? | | | 1. Yes  2. No | |
| 401 | | | Are you currently an alcohol drinker? | | | 1. Yes  2. No | |
